# Cost and environmental analysis and optimization of a new and green three-level waste heat recovery-based cogeneration cycle: A comparative study

**DOI:** 10.1016/j.heliyon.2024.e29087

**Published:** 2024-04-04

**Authors:** Hima Nikafshan Rad, Amir Ghasemi, Mohammad Marefati

**Affiliations:** a School of Information and Communication Technology, Griffith University, Nathan, QLD, Australia; b Department of Civil Engineering, School of Engineering, Monash University, Clayton Campus, Melbourne, Australia; c SavvyScience Tech Pty Ltd., Melbourne, VIC, 3752, Australia; d Department of Energy Engineering, Faculty of Natural Resources and Environment, Science and Research Branch, Islamic Azad University, Tehran, Iran

**Keywords:** *Three-level waste heat recovery*, *Cogeneration power/cooling system*, *Organic rankine cycle*, *Ejection refrigeration cycle*, *Comparative study*, *Environmental analysis*

## Abstract

Effective and maximum utilization of waste heat from industrial processes and fossil plants can improve thermodynamic performance and declined the environmental impacts of waste heat discharge to the atmosphere. Here, the multi-aspect assessment and optimization of a novel cogeneration power and cooling load cycle (CPCC) is developed. The considered cogeneration process is designed under a three-level waste heat recovery process consisting of an ORC (organic Rankine cycle) unit and an ejection-based refrigeration process. Thermodynamic performance, cost feasibility and environmental assessments of the suggested process have been comprehensively evaluated and discussed. A two-objective optimization is developed to minimize the total cost and maximize the exergy efficiency. Moreover, the comprehensive CPCC behavior is compared with a reference system (a single-level recovery/ORC process and a compression-based refrigeration process). The performance of the considered CPCC is also examined under various environmentally compatible refrigerants. The environmental analysis is based on two indicators (i.e., life cycle-climate performance and total equivalent-warming impacts). Due to the multi-level recovery of waste heat, the environmental impacts of emitting waste heat into the environment are significantly reduced. The outcomes revealed that the R1234/yf is considered as the most suitable refrigerant that can causes to optimum achievements for both systems. The exergetic performance is improved by about 10.3% compared to that reference system, while the exergy destruction and total annual cost of the CPCC, respectively, are reduced by approximately 7.4% and 21.6% compared to the reference cycle. It was also found that about 11,640 tons of carbon dioxide can be reduced by using the ejector in the refrigeration process.

## Nomenclature

C: Cost (USD)
E: Energy rate (kWh)
EA: Environmental assessment index
$\dot{E}x$: Exergy rate (MW)
h: Specific enthalpy (kJ/kg)
i: Interest rate
Lk: Leakage rate
$\dot{m}$: Mass flow rate (kg/s)
N: Lifetime of the project (years)
P: Pressure (bar)
$\dot{Q}$: Heat transfer rate (MW)
S: Entropy rate (kJ)
T: Temperature (^°^C)
$\dot{W}$: Power/Work (MW)
$\dot{Z}$: Investment cost rate (USD/h)

**Greek symbols**
α: Refrigerant recovery rate
β: carbon intensity factor
ε: Exergy efficiency (%)
τ: Yearly running hours
φ: Maintenance factor

**Subscripts**
0: Dead condition
an: Annual
Comp: compressor
Cond: condenser
CT: cooling tower
d: destruction
DE: direct emission
EJC: ejector
EVP: evaporator
Gen: generator
#HE: heat exchanger
IDE: indirect emission
INV: investment
lm: log-mean difference
OP: operation
P: pump
p: primary
q: heat
ref: refrigerant
s: secondary
Sep: separator
S-Turb: steam turbine
to: total
w: power

**Abbreviations**
CGU: Cooling Load Generation Unit
CO_2_: Carbon Dioxide
COP: Coefficient of Performance
CPCC: Cogeneration Power/Cooling Cycle
CRF: Capital Recovery Factor
GWP: Global Warming Potential
HRS: Heat Recovery System
ODP: Ozone Depletion Potential
ORC: Organic Rankine Cycle
PGU: Power Generation Unit
RFU: Refrigeration Unit
VERC: Vapor Ejection Refrigeration Cycle

## 1 Introduction

The ever-increasing demands for energy all over the world due to rising living standards and industrial and social development have created problems in the field of sustainable and secure energy supply [1,2]. Based on this, energy scientists suggested that new energy systems should be exploited alongside traditional energy systems/power plants [3,4]. A new energy system/plant in order to be exploited and operated (in power plant and/or distributed generation scale) should have a well-founded capital cost and improve the thermodynamic performance [5,6]. Besides that, due to serious environmental concerns, new energy systems must be environmentally friendly and comply with international carbon laws [7,8].

According to international agreements, achieving neutral-carbon emissions is one of the ideas for sustainable development in today's societies [9,10]. However, over 80% of the society's energy requirements are met through fossil fuel-burned power plants, which results in a huge amount of carbon being released into the atmosphere [11,12]. In 2019, about 70% of the carbon emissions released into the atmosphere were related to the burning of fossil energies [13]. Indeed, burning these fuels in power plants, industrial processes, and engines leads to the generation of a huge amount of waste heat in their exhaust [14,15]. The direct release of exhaust's waste heat to the environment causes irreparable damage to the health of society and the ecosystem [16,17]. Based on this, the importance of heat recovery systems (HRSs) is revealed in order to use exhaust heat instead of wasting it [18]. Based on HRSs, the industrial processes/plants' waste heat can be effectively recovered and converted into useful energy in different cycles [19,20]. In addition to improving the process's efficiency, this can significantly reduce environmental concerns caused by fossil fuels [21,22].

HRSs can be implemented in accordance with ORC, Kalina cycles, different refrigeration cycles, thermoelectric generator, thermionic generator, water electrolysis cycles, etc., and convert waste heat into useful energy (e.g., electricity, heating load, cooling load, hydrogen fuel, etc.) [23,24]. ORCs are one of the most popular power generation cycles that can work based on HRSs [25]. The boiling point of an organic fluid is lower than water, which allows evaporation at a lower temperature (the suitability of ORC for working with low-temperature heat sources) [26]. The multi-criteria evaluation of the ORC units has been reported in many literatures, which indicates the suitability of ORC for waste heat recovery usages. The ORC units can be integrated with solar thermal collectors, geothermal sources, biomass gasification cycles, waste heat from industrial processes and fossil-fueled plants, various types of fuel cells, etc. [27,28]. Reshaeel et al. [29] found that the highest waste heat recovery rate was obtained when using R245fa as an organic fluid. Köse et al. [30] observed that the ORC (under n-Pentane) could provide better thermodynamic and environmental performances compared to Kalina cycle. However, the Kalina cycle had a shorter payback period of approximately 0.1 years. Lan et al. [31] found that integrating an ORC unit with a thermoelectric generator could effectively improve the waste heat recovery rate. Moreover, such an integrated system was able to provide considerable fuel savings compared to a single ORC unit or single thermoelectric generator.

Although fossil power plants release a significant amount of carbon dioxide (CO_2_) into the atmosphere, the robustness of the power grids depends on these plants. Accordingly, waste heat recovery technologies of fossil power plants and reduction of CO_2_ emissions can be a useful environmental and thermodynamic approach [32]. In Ref. [33], a post-combustion CO_2_ capture cycle coupled with an ORC unit was developed to attain a high CO_2_ capture ratio and low energy utilization. The results showed that the carbon capture rate and the ratio of electricity production to consumption were 97.5% and 49.5%, sequentially. Saedi et al. [34] provided the feasibility valuation of a power plant integrated with an ORC unit to produce the supplementary thermal power in a greenhouse. The increase in greenhouse temperature is reported to be about 7.5 ^°^C. The payback period for that project was reported to be nearly 5 years. By developing a data-based model and efficient predictive control approach, Shi et al. [35] indicated that an ORC unit is significantly distinct in waste heat recovery.

Such a model could realize the reasonable adjustment of the HRS based on an ORC unit. Wang et al. [36] proposed the multi-criteria assessment of a hybrid energy cycle (a gas turbine, a solid oxide fuel cell, and an ORC unit) to build a green HRS in order to power the ships. They reported that focusing on enhancing the heat exchanging could decline the initial capital. Accordingly, adding cascade heat exchangers in the ORC unit as a multi-level recovery of waste heat to enhance the recovery rate can improve thermodynamic performance and mitigate the environmental impacts.

The refrigeration process is another thermodynamic cycle that can efficiently produce a cooling load by effectively recovering the waste heat. The ejection and compression refrigeration processes are two common approaches to generate cooling load through waste heat recovery technology [37]. In the ejection-based refrigeration process, instead of a compressor, an ejector is employed that does not need to consume electricity. Accordingly, such a process can notably lower the electrical energy utilization of the refrigeration cycle, which consequently improves the thermodynamic efficiency [38]. In addition, in the compression-based refrigeration process, an expansion valve is employed, which causes the loss of a part of the cooling load during the expansion process [39,40].

The ejection-based refrigeration process can offer better efficiency due to the reduction of thermodynamic losses, and its operation is relatively simpler [41]. Bai et al. [42] developed a modified parallel compression Transcritical CO_2_-refrigeration based on a sub-cooler and an ejector to improve the thermodynamic behavior considering the effects of the different parameters (such as discharge and flash pressures and cooler outlet and evaporation temperatures). They reported that the use of that system could drop the optimum flash pressure by up to 33% and compressor displacement by approximately 11.5%. Moreover, such a cycle was able to improve the COP (coefficient of performance) value by about 16%.

Lu et al. [43] proposed an ejector-expansion refrigeration process to establish a −50 ^°^C low-temperature freezer based on the experimental and exegetic methods. They found that the compression ratio of the ejector-based refrigeration process could be reduced by ∼16% compared to the common single-level process. Walid Faruque et al. [44] examined the thermal operation of a cascade refrigeration process under an ejector expansion vapor compression cycle and a flash tank. The findings indicated that refrigeration process could improve the COP value and exergetic behavior (at −60 ^°^C). Yang et al. [45] observed that the COP for a Transcritical CO_2_ ejection/expansion two-level compression refrigeration process was about 2.3-fold higher compared to a Transcritical CO_2_ two-level compression refrigeration process.

According to the literature, energy systems based on more than one output useful product can enhance the thermodynamic efficiency of the energy process by improving the waste heat recovery rate. It can increase the reliability, stability, and popularity of the plants [46]. A cogeneration electricity and cooling load process based on HRSs can be an efficient, modern and promising option [47]. However, due to increasing globalization and commercialization of such systems, it is needful to carry out detailed, comprehensive and comparative studies to highlight the advantages of the cogeneration electricity and cooling load process. A comprehensive analysis based on thermodynamic analysis, cost feasibility, and environmental assessment can provide a fruitful evaluation for a new proposed energy system.

The hybrid energy plants based on an ORC unit and refrigeration cycle had been reported in some publications. Liu et al. [48] proposed an integrated system for high-value utilization of waste heat based on a double-level absorption refrigeration cycle and an ORC unit considering the multi-scenario scheduling optimization. They found that the improvement rate of the operational stability of waste heat utilization was 69%. Naquash et al. [49] performed the energy, exergy, environmental, and economic analysis of an coupled hydrogen liquefaction coupled with an ORC unit and a absorption refrigeration cycle. The specific energy value of 6700 Wh per kg was required for entire process. Ashwni et al. [50] designed a combined plant (an ORC unit and a dual evaporator vapor compression refrigeration). They reported that the optimum exergy efficiency (∼11%), COP value (0.38), and total cost (∼33.1 k USD) were obtained under butane as working fluid.

Shi and Asgari [51] developed a CPCC based on a dual-pressure ORC unit and an ejection-based refrigeration process integrated with an energy storage cycle. The optimal total unit cost was 13.5 $ per GJ. Tashtoush and R. Algharbawi [52] developed a hybrid solar ejector cooling with ORC unit considering the variable geometry of the ejector to accommodate the growth in heat. They found that an enhancement of 78% of the COP value was achieved. Moreover, the total system efficiency and reduction rate of CO_2_ emissions were 11.35% and 3.2 tons/kg, respectively. Yu et al. [53] reported that the perfluoropropane was the optimal operating fluid for a CPCC based on an ORC unit and a ejector-based refrigeration system.

Elakhdar et al. [54] proposed a solar heat-driven CPCC based on an ORC unit and ejector. The COP value of that system was 0.17. However, the value of COP could be improved by reducing the ejector area ratio and enhancing the turbine extraction ratio. Sanaye and Refahi [55] developed a combined ORC unit and ejector-based refrigeration cycle to transformer and space cooling of the gas engines applications. The additional heat of the engine's exhaust was utilized in the ORC unit to produce power. They observed that the cooling capacity and COP value were nearly 271 kW and 0.33, respectively.

Wang et al. [56] presented a combined cooling and power system under the geothermal flash cycle, ORC, and ejector refrigeration cycle. For efficiency improvement, zeotropic mixtures were employed in ORC and refrigeration units. Total energy and exergy efficiencies were obtained 18.16% and 59.16%, respectively. Montazerinejad et al. [57] evaluated a solar-driven combined cooling, heating and power system under both conventional and advanced exergy analysis to see the effect of nanofluid inside the collector on heat transfer coefficient. They reported that even technological maturity will not be able to prohibit the triggered endogenous exergy.

To achieve the increasing globalization and commercialization of the cogeneration electricity and cooling processes, it is needful to carry out detailed, comprehensive and comparative studies to highlight the thermodynamic and environmental advantages. Effective and maximum use of waste heat can improve the thermodynamic performance and decline the environmental impacts. In this paper the multi-aspect assessment and optimization of a cogeneration electricity and cooling load process is developed. The considered cogeneration process is designed under a three-level waste heat recovery process based on an ORC unit and an ejection-based refrigeration process. Thermodynamic performance, cost feasibility and environmental investigations of the suggested process are comprehensively evaluated and discussed. A two-objective optimization is established to minimize the total cost and maximize the exergy efficiency. Moreover, the comprehensive CPCC behavior is compared with a reference system (a single-level recovery/ORC process and a compression-based refrigeration process). The CPCC performance is also examined under various zero-ODP (ozone depletion potential) and low-GWP (global warming potential) refrigerants. Furthermore, the developed environmental analysis is based on two indicators: life cycle-climate performance and total equivalent-warming impacts. According to the literature [58], these two environmental analyzes can be the appropriate approaches to evaluate the environmental impacts of new generation energy systems. In summary, the advantages and innovations of the proposed process can be stated as:

• Improving the thermodynamic performance of the cogeneration electricity and cooling load cycle and reducing the environmental impacts of emitting waste heat into the atmosphere due to the multi-level recovery of waste heat;
• Self-sufficiency of the process in meeting the electricity requirements of electrical equipment and consequently reducing the system electricity cost;
• Improving the cooling capacity by reducing energy losses during the refrigeration cycle, consequently reducing the system operating cost.

In the next part of the article, the performance description and modeling of both proposed and reference systems are presented. System modeling is presented based on thermodynamic analysis, economic analysis, and environmental analysis. In addition, in the second section, the optimization algorithm is described. The third part is related to model validation. In the fourth part, the results obtained from the research are presented and discussed. In addition, a comparative analysis is presented in this section. The conclusions of the research are finally presented in the fifth section.

## 2 Performance description and modeling

**Fig. 1:**
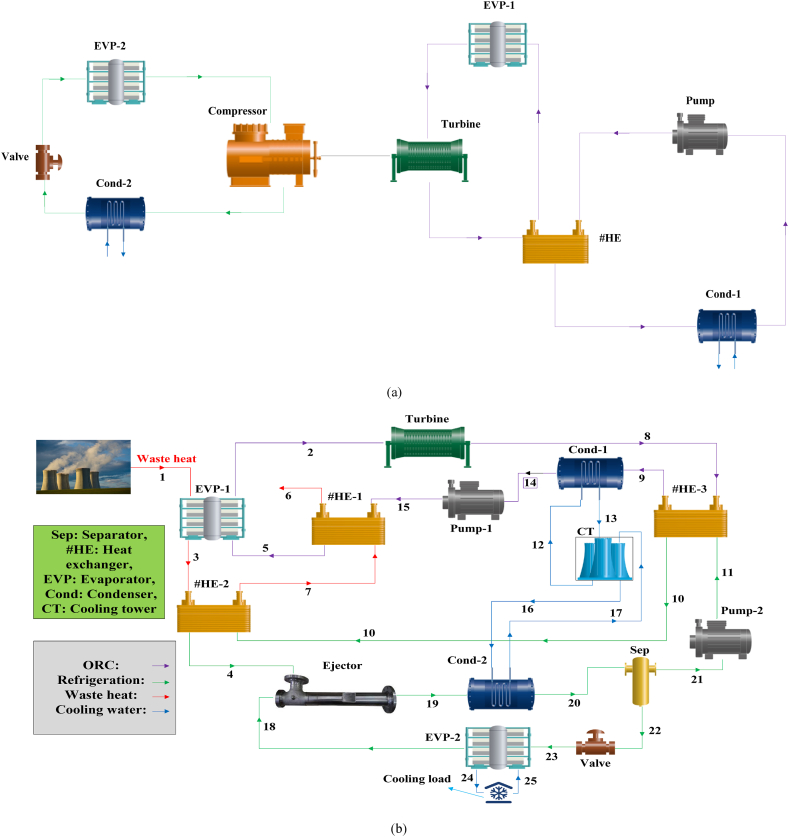
Schematic diagram and configuration of the reference system (a) and proposed CPCC (b).

The target of this article is to multi-criteria evaluate a novel waste heat recovery cycle to produce electricity and cooling load. For this purpose, it is assumed that low-temperature waste heat (at 120 ^°^C) is available. The CPCC performance has been evaluated under different refrigerants. In addition, a comprehensive comparison between the performances of the CPCC and the common cycle suggested in the literature is provided. Fig. 1(a and b) depicts the schematic diagram and configuration of the reference structure and suggested CPCC. Aspen HYSYS and MATLAB^@^ software were used to simulate and evaluate the CPCC. Further, the simulation equations are based on the Peng-Robinson equation, as reported in Ref. [59]. Unlike the common cycles introduced in the literature, which the refrigeration cycle is based on a compression refrigeration process, the proposed CPCC is based on an ejection refrigeration process.

As seen in Fig. 1, the planned CPCC is comprised of four main units: a waste heat system, a power generation unit (PGU), a refrigeration unit (RFU), and a cooling load generation unit (CGU). Indeed, in the proposed CPCC, the refrigeration and power units are based on a VERC (vapor ejection refrigeration cycle) and an ORC system, respectively. The PGU is comprised of a steam turbine (S-Turb), a pump (P), a condenser (Cond), and heat exchangers (#HE) to recovery of waste heat; while, the RFU is based on a Cond, an evaporator (EVP, to produce cold energy), a pump, and two heat exchangers. In the basic cycle introduced by the researchers, the heat recovery process is based on one heat exchanger; while, in the proposed CPCC, three heat exchangers are employed to recovery of waste heat to improve the heat recovery level.

A cooling tower (CT) is employed in the CGU to supply condensed water to the PGU. Finally, instead of using the expansion valve and compressor (Comp), an ejector (EJC) has been employed. According to the literature [60], using an EJC can significantly reduce energy consumption and losses. Besides that, the produced electrical energy can meet the electrical requirements of all the installed electric equipment in the proposed energy system. This can bring both thermodynamic improvement and reduce the electricity cost of the planned CPCC. The embedded heat exchanger at the border of the PGU and RFU units heats the separator (Sep) outlet state to decline the thermal function of the Cond-1. This can diminish the refrigeration cycle's operating cost. Therefore, the CPCC can improve the thermodynamic performance of the cogeneration electricity and cooling load cycle and reducing the environmental impacts due to the multi-level heat recovery. Moreover, by improving the cooling capacity (by reducing energy losses during the refrigeration cycle) the operating cost was declined. As mentioned, the CPCC performance is examined under different low-GWP refrigerants (hydrofluoroolefins). These refrigerants are: R1234/yf, R1336/mzz/E, R1225/zc, R1216, and R1354/mzy/E. The physical characteristics of these refrigerants are tabulated in Table 1. As deminstrated, the highest GWP value is related to the R1336/mzz/E.

**Table 1:** Physical characteristic of the considered refrigerants.

Refrigerant	Boiling point, ^°^C	Chemical abstracts service number	GWP
R1234/yf	−29.0	754/12/1	4.00
R1336/mzz/E	7.5	66711/86/2	18.00
R1225/zc	−21.03	690/27/7	4.30
R1216	−29.6	116/15/4	8.70
R1354/mzy/E	14.36	791616/87/0	–

### 2.1 Thermodynamic analysis

Thermodynamic analysis is performed based on the simulation to determine the thermophysical characteristics of the inlet/outlet streams of the components. According to the first law of thermodynamics, the mass/energy balance equations (Equation (1)), and according to the thermodynamics second law, the exergy balance equations (Equation (2)) for all components are written as [61]:

(1) $$\left\{ \begin{array}{c} \sum {\dot{m}}_{inlet}=\sum {\dot{m}}_{outlet}\\ \sum {\dot{m}}_{inlet}h_{inlet}+\dot{Q}=\sum {\dot{m}}_{outlet}h_{outlet}+\dot{W} \end{array}\right.$$

and

(2) $$\sum {\dot{Ex}}_{inlet}+{\dot{Ex}}_{q}=\sum {\dot{Ex}}_{outlet}+{\dot{Ex}}_{w}+{\dot{Ex}}_{d}$$

where, $\dot{m}$ is the mass flow rate, h is the specific enthalpy, $\dot{W}$ refer to the work, and $\dot{Q}$ denotes the heat transfer rate. In addition, $\dot{E}x$ and ${\dot{E}x}_{d}$ are the exergy flow and destructed exergy of a stream. The work produced via an expander (turbine) can be expressed by Equation (3) [62]:

(3) $${\dot{W}}_{Turb}=\left(h_{inlet}-h_{outlet}\right).{\dot{m}}_{inlet}$$

in addition, the energy utilized via the pump/compressor can be estimated by Equation (4) [63,64]:

(4) $${\dot{W}}_{Pump/Comp}={\dot{m}}_{inlet}\times \left(h_{outlet}-h_{inlet}\right)$$

Moreover, the heat transfer rate in a Cond is expressed by Equation (5):

(5) $${\dot{Q}}_{Cond}={\dot{m}}_{inlet}\times \left(h_{inlet}-h_{outlet}\right)$$

therefore, it is possible to obtain the electricity production and consumption rates, the net electricity, and the energy efficiency of the considered CPCC. Besides that, based on the second law and using exergy evaluation, by determining the irreversibility and inefficiencies, it is possible to identify the solutions to achieve optimal thermodynamic performance. A stream's exergy is based on chemical, physical, kinetic and potential exergy flows. From Ref. [46], the last two terms can be ignored in the exergy investigations of the energy system. Further, no chemical reaction is performed in the CPCC. Accordingly, the exergy rate for each inlet/outlet flow can be written as Equation (6) [65]:

(6) $${\dot{Ex}}_{i}=\left[\left(h-h_{0}\right)-T_{0}\times \left(s-s_{0}\right)\right]\times {\dot{m}}_{i}$$

Here, subscript 0 expresses the dead condition. In equation (2), ${\dot{E}x}_{q}$ represents heat exergy, which represents the maximum converted work based on the Carnot cycle, as Equation (7) [66]:

(7) $${\dot{Ex}}_{q}=\dot{Q}\times \left(1-\frac{T_{0}}{T_{lm}}\right)$$

where, $T_{lm}$ refers to the log-mean temperature difference. Exergy destruction is an obligatory parameter in the exergy investigation, measuring the resource degradation [67]. The destructed exergy in the overall CPCC is the sum of the destructed exergies in all components. Table 2 presents the equations to estimate the destructed exergy rates.

**Table 2:** The equations to estimate the destructed exergy rates.

Component	Exergy destruction	Component	Exergy destruction
Turbine	${\dot{E}x}_{2}-{\dot{E}x}_{8}-{\dot{W}}_{Turb}$	#HE-1	${\dot{E}x}_{7}+{\dot{E}x}_{15}-{\dot{E}x}_{5}-{\dot{E}x}_{6}$
EVP-1	${\dot{E}x}_{1}+{\dot{E}x}_{5}-{\dot{E}x}_{2}-{\dot{E}x}_{3}$	#HE-2	${\dot{E}x}_{3}+{\dot{E}x}_{10}-{\dot{E}x}_{4}-{\dot{E}x}_{7}$
EVP-2	${\dot{E}x}_{23}+{\dot{E}x}_{25}-{\dot{E}x}_{18}-{\dot{E}x}_{24}$	#HE-3	${\dot{E}x}_{8}+{\dot{E}x}_{11}-{\dot{E}x}_{9}-{\dot{E}x}_{10}$
Pump-1	${\dot{E}x}_{14}-{\dot{E}x}_{15}+{\dot{W}}_{P-1}$	Ejector	${\dot{E}x}_{4}+{\dot{E}x}_{18}-{\dot{E}x}_{19}$
Pump-2	${\dot{E}x}_{21}-{\dot{E}x}_{11}+{\dot{W}}_{P-2}$	Separator	${\dot{E}x}_{20}-{\dot{E}x}_{21}-{\dot{E}x}_{22}$
Cond-1	${\dot{E}x}_{9}+{\dot{E}x}_{12}-{\dot{E}x}_{13}-{\dot{E}x}_{14}$	Valve	${\dot{E}x}_{22}-{\dot{E}x}_{23}$
Cond-2	${\dot{E}x}_{16}+{\dot{E}x}_{19}-{\dot{E}x}_{17}-{\dot{E}x}_{20}$		

In the proposed CPCC, the ejection refrigeration process has been replaced by the compression refrigeration process to improve the refrigeration process performance. Accordingly, it is necessary to consider the modeling of the ejector. The mass entrainment ratio (MER) is an serious factor in the modeling of the ejector refrigeration process, which expresses the ratio of the secondary flow mass flow rate (${\dot{m}}_{s}$) to that of the primary flow (${\dot{m}}_{p}$), as Equation (8) [41]:

(8) $$MER=\frac{{\dot{m}}_{s}}{{\dot{m}}_{p}}$$

**Fig. 2:**
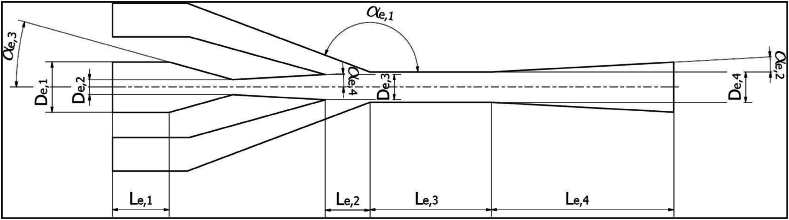
Schematic of the internal geometry of the ejector, adopted from Ref. [68].

The cooling requirements, working fluid selection, and ejector geometry are the essential parameters in modeling the ejection refrigeration process. The CPCC performance is examined under different low-GWP and zero-ODP refrigerants. The schematic of the internal geometry of the ejector is portrayed in Fig. 2, which is adopted from Ref. [68]. The main dimensions of the ejector are available in the same reference. Labeled parameters in the ejector schematic represent key geometrical parameters. These parameters have a significant effect on the mixing, entrainment, and expansion processes [69]. Accordingly, the value of MER used in the present research is based on the optimized reports by Galindo et al. [68]. Evaporating and condensing temperatures were considered equal to 13 and 40 ^°^C, respectively.

To evaluate the refrigeration system's operation, the COP is employed. For the CPCC, the value of COP can be defined by Equation (9) [32]:

(9) $$\text{COP}=\frac{{\dot{Q}}_{EVP}}{{\dot{W}}_{P-2}+{\dot{Q}}_{Gen}}$$

where, ${\dot{Q}}_{EVP}$ is the evaporator's thermal duty, ${\dot{W}}_{P}$ refers to the pump (P-2) work, and ${\dot{Q}}_{Gen}$ denotes the thermal duty of the generator (here, #HE-2).

### 2.2 Economic analysis

The total cost of the proposed CPCC is the sum of operating cost and equipment costs. In this regard, the overall investment cost is balanced using the capital recovery factor (CRF). This factor (a function of the interest rate (i) and the lifetime of the project (N)), is determined by Equation (10) [70]:

(10) $$\text{CRF}=\frac{i.{\left(i+1\right)}^{N}}{{\left(i+1\right)}^{N}-1}$$

accordingly, the CPCC total cost rate can be determined by Equation (11) [71]:

(11) $${\dot{Z}}_{\text{tot}}=\sum_{k}{\dot{Z}}_{k}=\sum_{k}\frac{{\dot{Z}}_{k,CI}\times \varphi \times \text{CRF}}{\tau }$$

where, ${\dot{Z}}_{CI}$, τ, and φ are the capital investment, total working hours (7200 h), and maintenance factor (1.06). The initial capital equations of the different components are presented in Table 3.

**Table 3:** Capital investment equations for the different components [[72], [73], [74]].

Component	Investment cost equation
Turbine	$Z_{Turb}=4405\times {\dot{W}}_{Turb}^{0.89}$
Heat exchanger	$Z_{\#HE}=3\times 10^{4}+750\times A_{\#HE}^{0.81}$
Ejector	$Z_{Ejec}=1.59\times 10^{4}\times {\dot{m}}_{s}\times {\left({T/P}_{inlet}\right)}^{0.05}\times P_{outlet}^{-0.75}$
Condenser	$Z_{Cond}=3\times 10^{4}+750\times A_{Cond}^{0.81}$
Pump	$Z_{P}=3.54\times 10^{3}\times {\dot{W}}_{P}^{0.71}$
Evaporator	$Z_{EVP}=3\times 10^{4}+750\times A_{EVP}^{0.81}$

Note: The capital investment costs of the ejector and valve can be neglected since their contribution to the total cost is so minor [57,69].

### 2.3 Environmental analysis

An innovative energy system designed under a new configuration and structure can be reliable and sustainable when, in addition to improving the thermodynamic operation and reasonable investment cost, it should be able to reduce the environmental impacts caused by the operation of the energy process. Thus, it is necessary to examine and discuss the environmental analysis of the plant/energy system [4]. Even though different low-GWP and zero-ODP refrigerants are utilized in the present cycle, there is a possibility of refrigerant leakage during the energy cycle operation [41]. Accordingly, the environmental analysis of the proposed CPCC is necessary.

The developed environmental analysis (EA) in this article is based on two indicators: 1) Total equivalent-warming impacts (EA-1) and 2) Life cycle-climate performance (EA-2). Both of these indicators can be suitable for the environmental analysis, because they represent the climate change and carbon emission [75]. From the literature [76,77], EA-1 indicates the effect of refrigerant leakage on the atmosphere, which is estimated by Equation (12):

(12) $$EA_{1}={\dot{m}}_{ref}.GWP.\left[\left(n\times Lk_{an}\right)+\left(1-\alpha_{re\text{cov}ery}\right)\right]+\left(n.E_{net}.\beta \right)$$

where, ${\dot{m}}_{ref}$, n, ${Lk}_{an}$, and $E_{net}$ refer to the refrigerant flow rate, equipment lifetime, refrigerant annual leakage rate, and annual energy rate, respectively. Further, $\alpha_{recovery}$ and β denote the refrigerant recovery rate and carbon intensity factor, respectively. Equation (12) describes the aggregate of the refrigerant leakage rate and the CO_2_ release rate through electrical components. Indeed, with the enhancement in electric energy consumption by the energy system, the value of EA-1 will increases.

EA-2 is another indicator used for the environmental assessment of the proposed CPCC. This index exhibits the effect of refrigerant use during the life cycle of the energy process. The EA-2 is estimated by Equation (13) [77]:

(13) $$EA_{2}=EA_{2,DE}+EA_{2,IDE}$$

here, subscripts *DE* and *IDE* denote the direct emission and indirect emission, respectively. Direct emissions are the sum of emissions from refrigerant leakage, refrigerant leakage during service operations and unexpected events, end-of-life refrigerant (when the refrigerant is lost), refrigerant transportation and production, and by-products from refrigerant emissions into the atmosphere. In addition, indirect emissions are the sum of emissions from energy consumption due to transportation of equipment, energy consumption during the lifetime of the project, and energy consumed during the construction of equipment and refrigerant. Equation (13) indicates that EA-2 considers the effect of both direct and indirect emissions of refrigerant and electrical equipment.

The examination of the CPCC overall performance is based on obtains the overall exergy efficiency (ε), total destructed exergy (${\dot{E}x}_{d,tot}$) and total system cost ($C_{tot}$), which are determined by equations (14), (15), (16)):

(14) $$\varepsilon =\frac{{\dot{W}}_{net}+\dot{E}x_{outlet}}{\dot{E}x_{inlet}+\dot{E}x_{q}+\dot{E}x_{w}}$$

(15) $$\dot{E}x_{d,tot}=\sum_{k}\dot{E}x_{d,k}$$

(16) $$C_{tot}=C_{INV}+C_{OP}$$

Also, the efficiency of the ORC unit is determined by Equation (17) [78]:

(17) $$\eta_{ORC}=\frac{{\dot{W}}_{Turb}-{\dot{W}}_{Pump}}{{\dot{Q}}_{EVP}}\times 100$$

### 2.4 Optimization algorithm

To achieve optimum operation of the proposed CPCC, optimum outcomes can be acceded via applying optimization algorithms and finding optimum design operation. In this study, the maximum of the exergy efficiency and the minimum of the total cost under a two-objective optimization algorithm are determined. According to literature, genetic algorithm can be employed to optimize energy systems. This algorithm is based on the evolutionary trend, expressing the optimal concept of survival. According to the evolutionary process of the genetic algorithm, at each stage, the strongest individuals become stronger, and the weakest individuals are left out of the process [79]. Moreover, the Non-dominated Sorting Genetic Algorithm-II procedure was employed to two-objective optimization. Based on this procedure, the foremost responses were selected based on the Pareto frontier [[80], [81], [82]]. This procedure is utilized to identify whether the considered response is the foremost one.

Accordingly, the suitable Pareto frontier response is determined according to an appropriate decision-making process [[83], [84], [85]]. Among some techniques suggested in the publications, the optimization established here is based on the LINMAP decision model. This technique is suitable for determining the weight of criteria and sub-criteria and ranking options. According to the LINMAP method, the optimal solution has the smallest distance from the ideal point [86]. For optimization, several independent variables that are likely to have the greatest influence on the system performance are selected as the constraints of the optimization problem. The independent optimization variables and their bounds are tabulated in Table 4. Further, the optimization objective function is written by Equation (18):

(18) $$f=\mathrm{Max}\;\left(\varepsilon ,1-C_{tot}\right)$$

**Table 4:** Independent optimization variables and their bounds.

Variable	Lower bound	Upper bound
Turbine outlet temperature (^°^C)	60	120
Refrigerant flow rate (kg/s)	1.2	3.2
Turbine inlet pressure (bar)	12	18
Cold temperature (^°^C)	6	12

To simulate the proposed CPCC, it is assumed that [53,68,87]:

• The proposed CPCC works in stability conditions;
• The low-temperature waste heat (at 120 ^°^C) is available;
• Heat losses and pressure drops in pipelines and heat exchangers are ignored;
• The model of the primary and secondary flows of the expansion process is introduced by an isentropic efficiency;
• The heat transfer between the equipment and the environment was negligible;
• The primary mass flow rate via the nozzle is at choking conditions;
• Both the primary and secondary flows are directed the ejector in a saturated vapor phase.

**Fig. 3:**
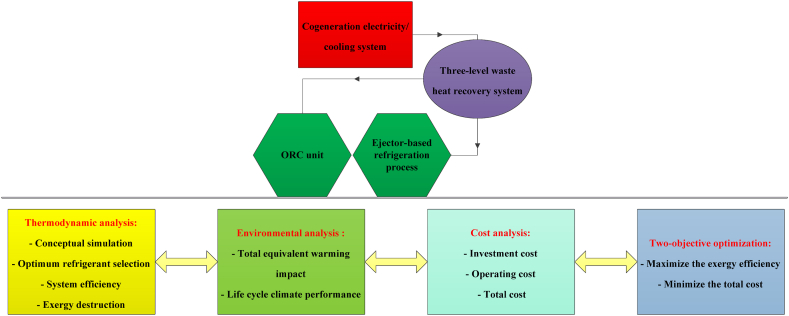
Analysis process of the proposed CPCC.

Other design and optimization conditions are stated in Table 5. Moreover, Fig. 3 shows how to analyze the proposed CPCC.

**Table 5:** Design and optimization conditions [53,88,89].

Parameter	Value	Parameter	Value
Reference pressure	1.013 bar	Maintenance factor	1.06
Reference temperature	25 ^°^C	Nozzle efficiency	85%
Refrigerant flow rate	2.5 kg/s	Mixing efficiency	90%
Turbine outlet temperature	100 ^°^C	Diffuser efficiency	85%
Turbine inlet pressure	16 bar	Isentropic efficiency	85%
Cold temperature	8 ^°^C	Pinch point temperature difference	5 ^°^C
EVP-1 pressure	32 bar	Yearly running hours	7200 h
EVP-1 temperature	150 ^°^C	Population size	2000
EVP-2 pressure	5 bar	Crossover probability	0.8
EVP-2 temperature	40 ^°^C	Stop generation	50
Mass entrainment ratio	0.418	Number of elites	100

## 3 Model validation

To confirm the simulation and modeling in this article, the provided models for the main units of the CPCC (ORC and RFU units as well as the ejector model) were compared with the data in the similar works.

The validation of the ORC model is according to the reported process conditions in Ref. [90]. T. White et al. [90] developed and compared an ORC unit based on a two-phase cascaded cycle for waste-heat recovery purposes. They evaluated optimization studies for three different heat source temperatures. The ORC unit was comprised of an EVP, a turbine, a pump and a Cond. The operating fluid of the ORC unit and inlet temperature of the heat source were R245fa (with flow rate of 4 kg/s) and 250 ^°^C, respectively. They reported that the EVP temperature was ∼173.5 ^°^C. Other design conditions are in accordance with the same reference. Further, to validate the net power of the cycle and the first-law and second-law thermal efficiencies obtained from the modeling and reported in the literature were compared. Table 6 shows the comparison and validation for model of the ORC unit. The biggest difference observed for the validation results of the ORC unit was equal to ∼2.4%. The small difference exhibits the accuracy of the modeling, which indicates that the developed modeling for the ORC unit can be used to evaluate the proposed CPCC with reliable accuracy.

**Table 6:** Model validation results.

Parameter	Literature	Modeling	Difference
**ORC unit** [90]			
Inlet temperature of heat source	250 ^°^C	250 ^°^C	0.0%
Heat stream rate	4 kg/s	4 kg/s	0.0%
Pressure of heat/cold stream	1 bar	1 bar	0.0%
Temperature of evaporator	173.4 ^°^C	171.7 ^°^C	1.05%
Organic fluid	R245fa	R245fa	–
Net power	33.91 kW	34.72 kW	2.39%
First-law thermal efficiency	16.47%	16.75%	1.70%
Second -law thermal efficiency	52.61%	51.84%	1.48%
**Ejection process-based refrigeration unit** [68]			
Temperature of evaporating	13 ^°^C	13 ^°^C	0.0%
MER value	0.408	0.408	0.0%
Temperature of condensing	40 ^°^C	40 ^°^C	0.0%
Refrigerant	R1234/yf	R1234/yf	–
Pressure of mixed flow	10.19 bar	9.88 bar	3.13%
Pressure of primary flow	37.74 bar	36.72 bar	2.77%
Pressure of secondary flow	4.80 bar	4.69 bar	2.34%
**Ejector simulation** [91]			
Pressure of primary flow	35.14 bar	34.18 bar	2.81%
Pressure of secondary flow	4.8 0 bar	4.70 bar	2.13%
Pressure of ejector outlet flow	10.18 bar	9.88 bar	3.03%
Liquid volume fraction	0.1745%	0.1801%	3.21%

The validation of the RFU model (based on the ejection process) is according to the reported process conditions in Ref. [68]. Galindo et al. [68] proposed the numerical assessment of a solar-ejector refrigeration cycle from an efficiency maximization point of view. They optimized the ejector internal geometry via a CFD code. They assessed the operation of the refrigeration cycle under various refrigerants. However, only the results for R1234/yf refrigerant were considered for validation. The RFU was comprised of an EVP, a Cond, a generator, an ejector, etc. Moreover, the evaporating and condensing temperatures and MER value were considered equal to 13 ^°^C and 40 ^°^C, and 0.408, respectively. To validate the pressures of the mixed, primary, and secondary flows obtained from the modeling and reported in the literature were compared (see Table 6). The highest difference observed for the validation results of the RFU was equal to 3.13%.

As mentioned, the small difference exhibits the accuracy of the modeling. Moreover, the numerical model developed for the ejector in Aspen software was compared with the observed findings based on the CFD simulation [91]. In this regard, the pressures of the primary, secondary, and ejector outlet flows obtained from the Aspen simulation and reported results [91] were compared. Table 6 lists the results of the comparison. The biggest difference observed for the validation results of the ejector simulation was equal to ∼3.2%. The small difference exhibits the accuracy of the modeling, which indicates that the established simulation for the ejector can be employed to investigate the proposed CPCC with reliable accuracy.

## 4 Results and discussion

The observed results of the thermodynamic, cost and environmental analyzes of the proposed CPCC under different operational conditions have been discussed. In this regard, the CPCC performance has been assessed for different refrigerants to select the refrigerant with higher thermodynamic performance. In addition, a comprehensive comparison between the performances of the CPCC and the reported reference system in the literature is presented. The purpose of comparing the two systems is to distinguish the advantages of the CPCC compared to the reference cycle.

As mentioned, in the CPCC and the reference structure, the refrigeration is based on the ejection and compression processes, respectively. In addition to the difference in the refrigeration process in the aforementioned two systems, the reference system uses a single-level heat exchanger to recover waste heat, while the recovery of waste heat in the proposed CPCC is done under a three-level cycle. Note that, the simulation and analysis have been done for both systems in the current work and the comparison is based on the results of the research. In addition, the comparison is based on the performance of the systems under the most suitable refrigerant. In this regard, five different low-GWP and zero-ODP refrigerants were examined.

**Fig. 4:**
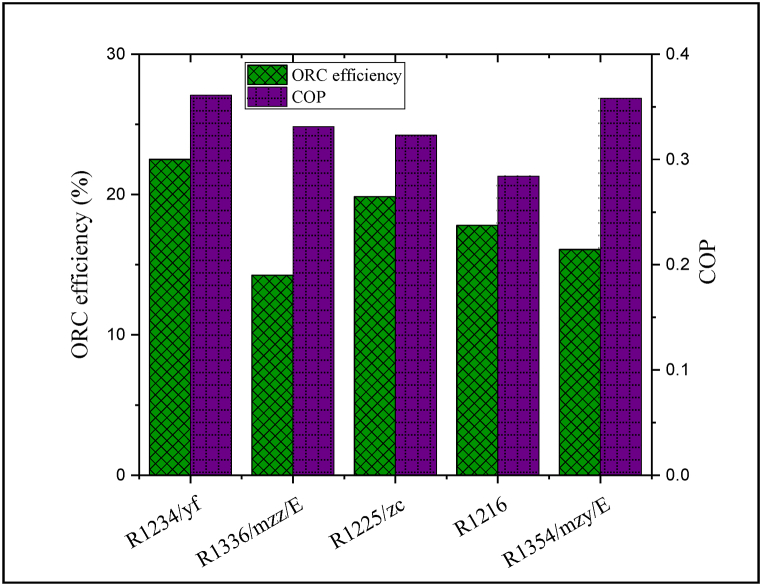
Obtained results for selecting the optimum refrigerant.

To select the most suitable refrigerant, the efficiency of the ORC unit and the COP value for the refrigeration process were calculated under all refrigerants. The obtained results for choosing the optimal refrigerant are plotted in Fig. 4. As depicted, R1336/mzz/E and R1234/yf refrigerants result in the lowest and highest efficiency of the ORC unit, respectively. Besides that, the lowest and highest values of COP are obtained under R1216 and R1234/yf refrigerants, respectively. Based on this, R1234/yf is considered as the most suitable refrigerant that can cause to optimum outcomes for both systems.

**Fig. 5:**
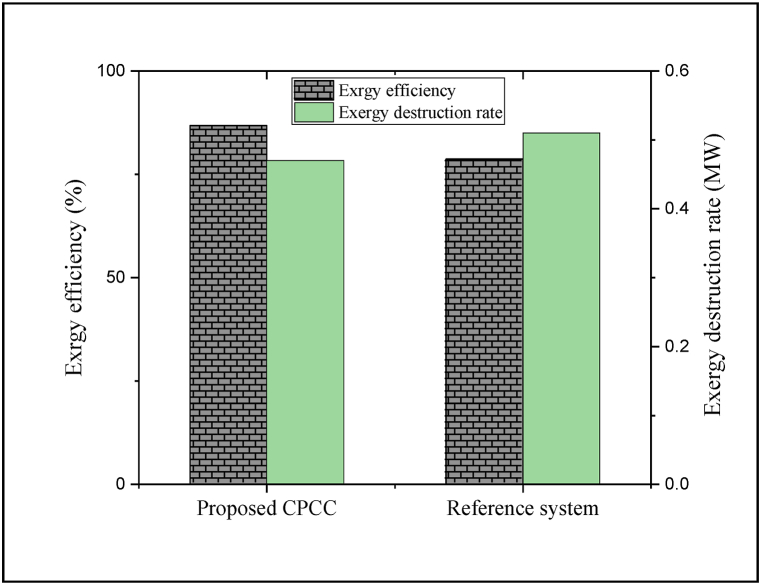
Comparison of the exergetic performance between the planned CPCC and the reference system.

Exergy analysis of the energy process expresses the efficiency that identifies the true measures to approach the ideal performance and determines the causes and locations of losses more powerfully compared to the energy examination. By using exergy examination, we can hope to improve the system's performance. According to this examination, the exergy criteria of different components and the entire CPCC are usually calculated [92]. The outcomes indicated that the CPCC can achieve an exergy efficiency of approximately 86.8%. Also, the overall exergy destruction was equal to ∼0.47 MW. The comparison of the exergetic performance between the planned CPCC and the reference system is portrayed in Fig. 5. The exergetic performance of the proposed CPCC is notably improved.

The exergetic efficiency of CPCC is improved by about 10.3%, while the exergy destruction rate is reduced by approximately 7.4%. The three-level recovery of waste heat in the CPCC compared to the single-level recovery in the reference system is one of the major reasons for reducing thermodynamic losses, reducing energy consumption, and consequently improving the exergetic performance. Determining the contribution of each component in exergy destruction can be effective in identifying thermodynamically inefficient components. Indeed, by improving the performance of thermodynamically inefficient components, the performance of the whole energy process can be enhanced.

**Fig. 6:**
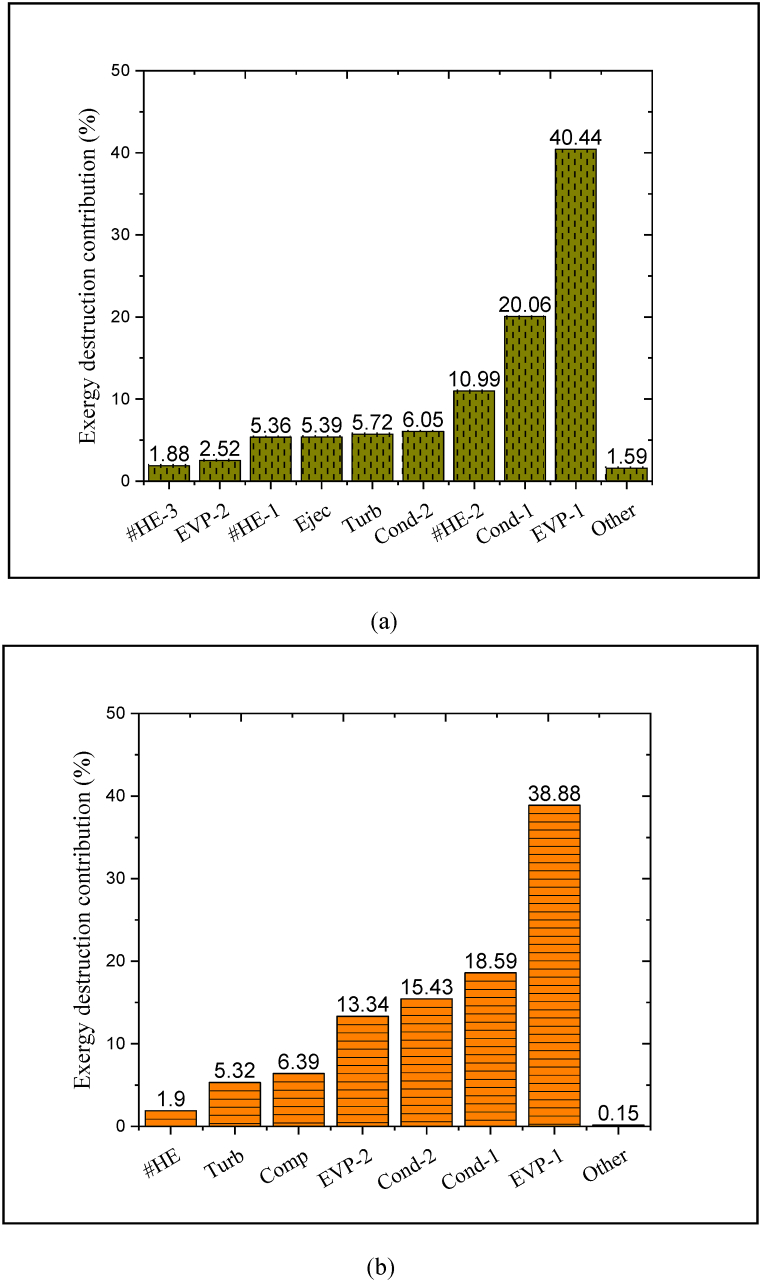
Contribution of each of the components of in the exergy destruction of the process, (a) proposed CPCC and (b) reference system.

Fig. 6(a and b) demonstrates the contribution of each of the components of both aforementioned systems in the exergy destruction. High thermodynamic losses and a large temperature difference between the inlet/outlet streams of a device are the major reasons for growing the destroyed exergy. For these reasons, in both the reference system and the proposed CPCC, heat exchangers (including heat exchanger, condenser, and evaporator) destroy more exergy compared to other components. Fig. 6(a) indicates that about 43% of the total exergy is destroyed by evaporators (EVP-1 and EVP-2); while, the embedded evaporators in the reference system destroy approximately 52.2% of the total exergy.

About 18% of the total exergy is destroyed through the three-level heat exchange (#HE-1, #HE-2, and #HE-3), while only ∼ 2% of the overall exergy of the reference structure is lost by the heat exchanger. In both desired systems, turbines destroy almost equal exergy (5.72 and 5.32%); because the turbine experiences almost the same thermodynamic conditions in both systems. Moreover, approximately 26.1% of the total exergy is destructed by condensers (Cond-1 and Cond-2); while, the embedded condensers in the reference system destroy almost 34% of the total exergy.

The thermal integration of the process causes the installed evaporator in the ORC unit (EVP-1) in the proposed CPCC destroy approximately 3.72% less exergy compared to that in the reference system. Finally, compared to the compressor (based on the compression process), the ejector (based on the ejection process) destroys almost 1% less exergy. Even though the compressor and ejector have almost the same exergy operation, unlike compressors, ejectors do not consume electrical energy, which reduces the electricity utilization of the energy process. It was also found that the exergy destruction rate of the whole refrigeration process in the CPCC is reduced by approximately 25.5% compared to that reference system. In other words, the share of the destructed exergy of the refrigeration process in the CPCC is about 7% less compared to that reference system. Therefore, replacing the ejector with the compressor can improve the cogeneration plant's performance.

**Fig. 7:**
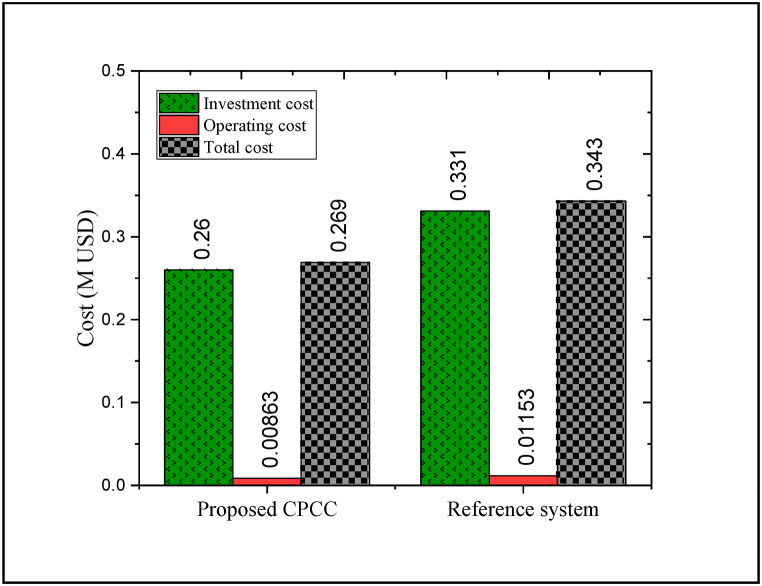
Comparison of the cost analysis between the proposed CPCC and the reference system.

The economic analysis of the considered CPCC is necessary to identify and establish its benefits for increasing the globalization of the energy system. As stated, three-level heat recovery and the replacing the ejector with the compressor reduce electricity consumption and economy. Based on this, the economic behaviors of both the offered CPCC and the reference system were compared. The outcomes of the economic analysis exhibited that the annual total cost estimate of the proposed CPCC is equal to 0.269 M USD, which is more than 96% related to the investment cost and about 4% related to the operating cost. The comparison of the cost analysis between the CPCC and the reference system is plotted in Fig. 7.

**Fig. 8:**
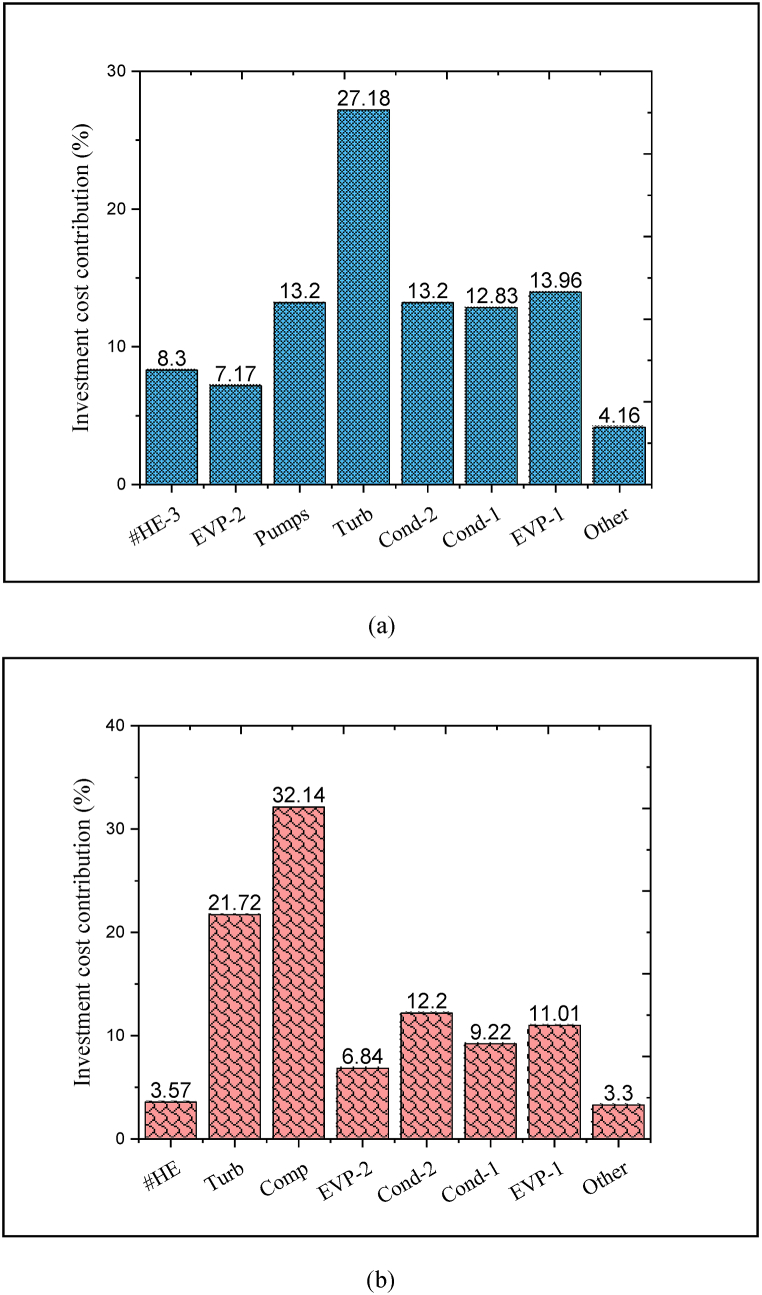
Contribution of each of the components in the investment cost, (a) proposed CPCC and (b) reference system.

As seen, the total annual cost of the CPCC can be declined by approximately 21.6% compared to the reference system. This is due to the reduction of energy losses and the improvement of the waste heat recovery rate, causes to a decrement in the investment cost of the equipment by almost 21.4%. Also, the annual operating cost of the CPCC is about 25.16% lower than the reference ones. Estimating the contribution of each component in the initial capital can be fruitful in identifying components with high economic cost. Indeed, by reducing the investment cost of high-cost components, the total cost was declined. Accordingly, Fig. 8(a and b) demonstrates the contribution of each of the components of both aforementioned systems in the investment cost. More than 32% of the investment of the reference structure is associated with the compressor (compression process), while this component has been replaced with an ejector in the CPCC. The investment of the ejector is a so small portion of the total capital cost of the proposed CPCC (less than 1%). Indeed, ∼0.1 M USD can be saved in the compressor cost. In addition to reducing the investment, this reduces the electrical energy consumption. In the proposed CPCC, due to the decrement of cooled water utilization and also the reduction of the thermal duties of the condensers, the investment costs of the condensers can be reduced by approximately 4.5% compared to the installed condensers in the reference system.

For the discussed reasons, the embedded turbines in both systems have approximately the same investment cost. However, three-level heat exchange requires nearly 175% more investment cost compared to single-level heat exchange. By the way, due to the removal of the compressor and the decrement in the energy utilization and thermal duties on the one hand and the improvement of the heat recovery rate and the self-sufficiency of the proposed CPCC in electricity supply on the other hand, the investment cost is 21.4% lower than that reference cycle. This can greatly contribute to the sustainability of the system in the direction of clean energy production. Moreover, Table 7 presents the investment costs of each component in the proposed CPCC and reference system. As seen, the proposed CPCC has a lower total investment cost compared to the reference system. The main reason for the high investment cost of the reference system compared to the proposed CPCC is the high cost of the compression process.

**Table 7:** Investment costs of each component in the proposed CPCC and reference system.

Proposed CPCC		Reference system	
Component	Investment cost 10^3^ USD	Component	Investment cost 10^3^ USD
Heat exchanger-3	21.580	Heat exchanger	11.816
Evaporator-2	18.642	Turbine	71.893
Pumps	34.320	Compressor	106.383
Turbine	70.668	Evaporator-2	22.640
Condenser-2	34.380	Condenser-2	40.382
Condenser-1	33.358	Condenser-1	30.518
Evaporator-1	36.296	Evaporator-1	36.443
Other components	10.816	Other components	10.923
Total investment cost	260.06	Total investment cost	330.998

**Fig. 9:**
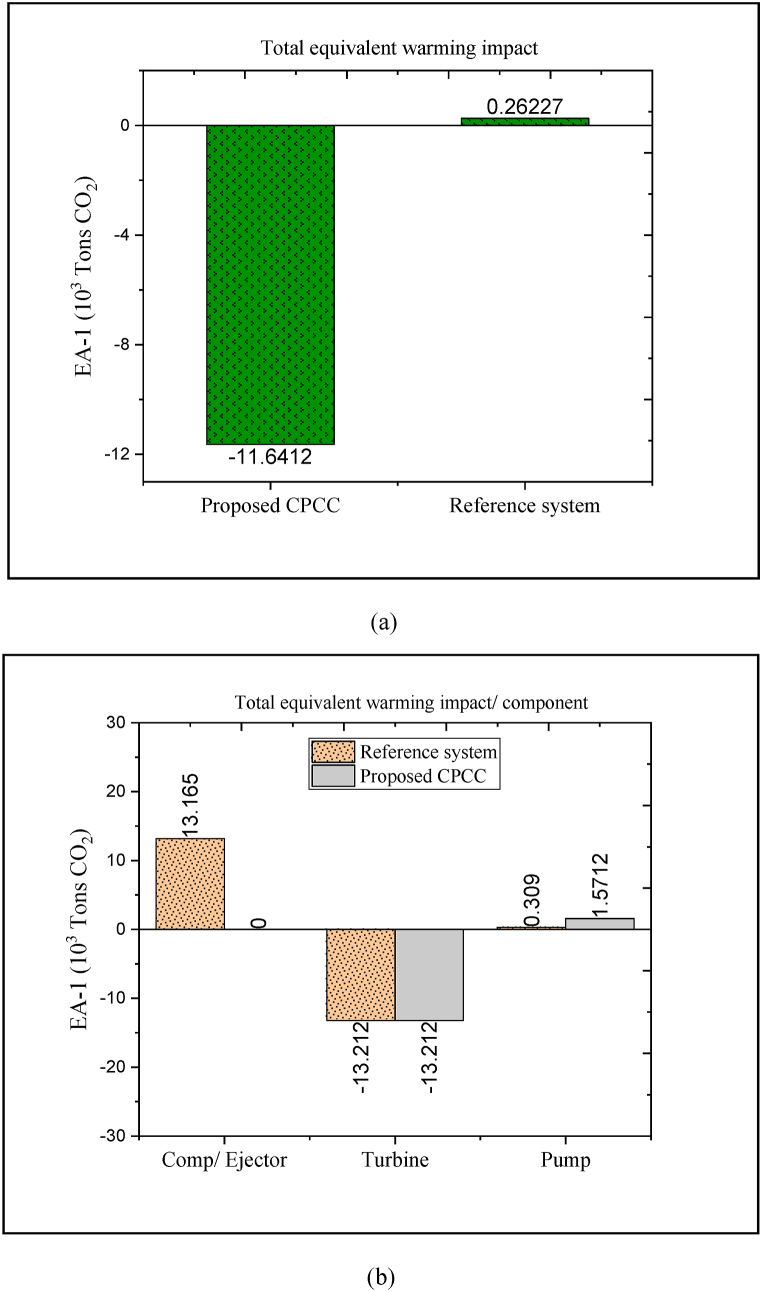
EA-1-based environmental assessment results of the proposed CPCC.

From the environmental point of view, two indicators of life cycle climate performance and total equivalent warming impact are determined to point out the potential cleanness and greenness of the proposed CPCC. These analyzes exhibit the offered CPCC impact on the environment during exploitation and energy production. EA-1 is the environmental assessment of the CPCC based on the value of GWP and the amount of electric energy consumed through electrical components. Fig. 9(a and b) shows the EA-1-based environmental assessment results of the CPCC, which is compared with the reference structure. Note that, the environmental assessment is based on equivalent released CO_2_. Moreover, in the plots, negative and positive values indicate the amounts of CO_2_ emission and CO_2_ production, respectively. To compare the environmental assessments of the CPCC and the reference structure, it is assumed that both processes operate under R1234/yf refrigerant. Accordingly, the difference in environmental performance is caused by the amount of electricity consumed. In other words, environmental analysis is considered from the electric energy consumption/production views.

The components that consume electrical energy, the amount of carbon dioxide emitted by them is considered positive, and the components that produce electricity (i.e., turbine) the amount of carbon dioxide emitted by them is considered negative. Fig. 9 (a) points out that the equivalent released CO_2_ from the proposed CPCC is remarkably lower compared to that reference system. Fig. 9 (b) indicates the contribution of each electrical component in equivalent CO_2_. It can be seen that in the reference system, a significant share of equivalent CO_2_ (more than 97%) is related to the compressor performance. However, in the proposed CPCC, an ejector has been employed instead of a compressor, which significantly reduces the value of the equivalent CO_2_. This exhibits that with the growth in the installation of electrical equipment and the consequent growth in the amount of energy consumption, the rate of carbon dioxide emission will increase.

However, by increasing the electrical energy generating equipment, the electricity output can be increased as well as the electricity utilization of the electrical components can be lowered. Indeed, the self-sufficiency of the energy system in supplying the required power causes a significant reduction in the emission of CO_2_. This fact is demonstrated in Fig. 9 (a) and 9 (b). In such a context, the energy system can become an environmentally friendly process. Fig. 9 (a) confirms that about 11,640 tons of carbon dioxide can be reduced by using the ejector in the refrigeration process. Therefore, the proposed CPCC can address better environmental performance than the reference cycle due to the use of low electricity consumption-equipment.

**Fig. 10:**
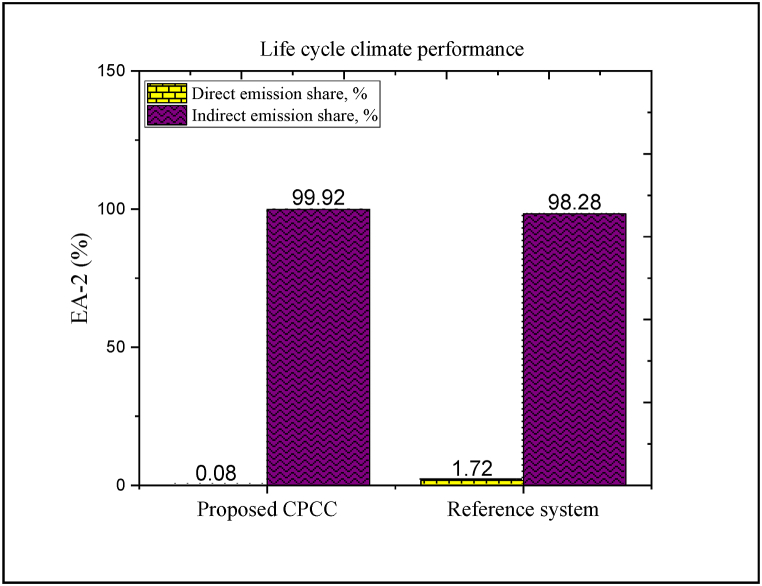
Results of the EA-2-based environmental analysis (Note: in the case of the proposed CPCC, indirect emissions indicate the reduction rate of carbon emissions).

The EA-2-based environmental analysis can provide a more accurate evaluation of carbon dioxide emissions compared to EA-1-based environmental analysis; because it considers the amount of carbon production directly and indirectly throughout the life of the project (including construction, operation, disposal, etc.). The outcomes of the EA-2-based environmental analysis are plotted in Fig. 10. Similarly, to develop the EA-2-based environmental analysis, it is assumed that both desired systems operate under R1234/yf refrigerant. The outcomes of the EA-2-based environmental analysis indicated that the amount of released CO_2_ that is directly produced is the same for both the CPCC and the reference structure. However, the share of released CO_2_, which is directly produced by the proposed CPCC, is far less compared to its share in the reference system (0.08% vs. 1.72%).

The reason for the difference in the share of released CO_2_, which is directly produced through the two aforementioned systems, is due to the replacement of the ejector instead of the compressor, which greatly reduces the consumption of electrical energy. As discussed under the EA-1-based environmental analysis, the proposed CPCC can reduce CO_2_ emissions compared to the reference system. According to the EA-2-based environmental analysis, the amount of the released CO_2_ that is indirectly produced by the reference system contributes more than 98% of the total released CO_2_; while the proposed CPCC not only does not indirectly release CO_2_, but also significantly reduces the amount of CO_2_ emissions. The amount of released CO_2_ that is indirectly produced through an energy system is originated by the electrical energy consumed by the electrical components (especially components with high-electricity consumption). Accordingly, the proposed CPCC can offer potential environmental benefits than the reference cycle.

The cause for the decrease in the amount of released CO_2_ that is indirectly produced through the proposed CPCC is that the electrical energy production rate is more than the electrical energy consumption rate of the system. This can reduce about 45-fold the amount of released CO_2_ that is indirectly produced by the reference system. The outcomes of the EA-2-based environmental analysis exhibit that reducing the employ of electricity-consuming components in the cycle and consequently the total electricity consumption are fruitful measures to improve the environmental performance.

**Fig. 11:**
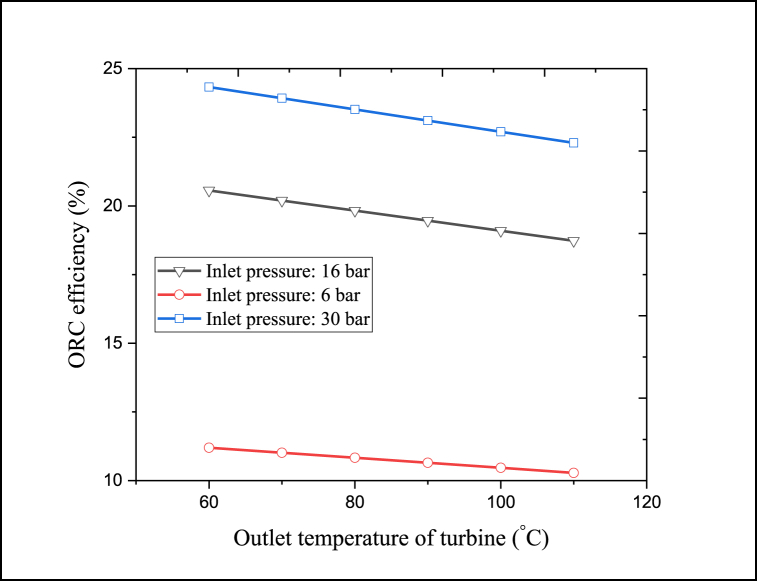
Changes in the ORC unit's energy efficiency with the turbine outlet temperature and inlet pressure.

The turbine outlet temperature and inlet pressure are among the factors that affect the ORC unit's performance and the CPCC performance. For this purpose, Fig. 11 depicts the changes in the ORC unit's energy efficiency with the turbine pressure and temperature. By increasing the turbine's inlet pressure (the turbine's outlet pressure is constant) the pressure difference between the inlet and exit flows of the turbine enhances, which increases the thermodynamic characteristics of the inlet stream to the turbine, improves the turbine's work production, and enhances the electricity output. While with the increase in turbine outlet temperature, thermal energy leaves the turbine without being converted into useful work. Accordingly, raising the turbine's outlet temperature decreases the electricity production rate. Therefore, raising the turbine inlet pressure or decreasing the outlet temperature enhances the electricity production rate. So, due to the constant EVP heat duty, the ORC thermal efficiency can be increased in such a context.

**Fig. 12:**
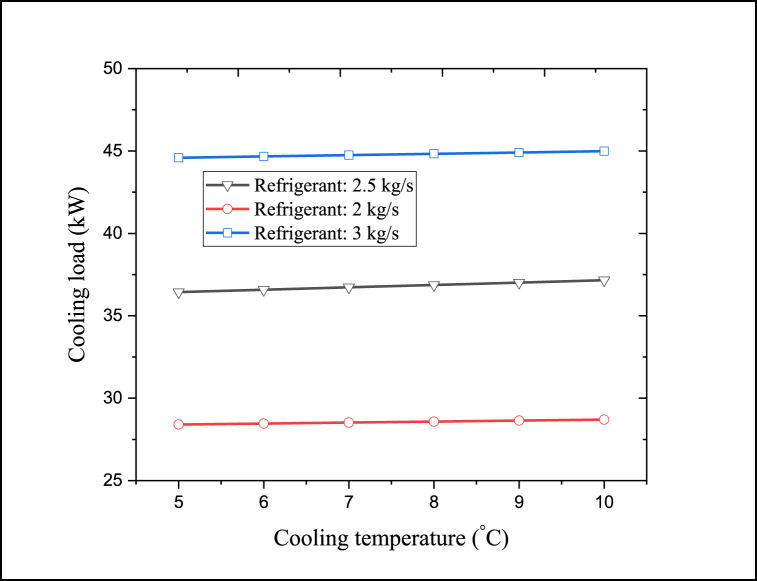
Variation of the cooling capacity of the proposed CPCC with the cooling temperature.

Cooling temperature is another parameter that affects the CPCC performance. Cooling temperature and cooling capacity have an indirect linear relationship. The variation of the cooling capacity of the proposed CPCC with the cooling temperature is plotted in Fig. 12. Obviously, to achieve cooler conditions, the cooling load production rate is reduced; because more input energy is consumed to reduce the temperature of the outlet flow of the refrigeration process.

Fig. 12 reveals that by elevating the cooling temperature by 5 ^°^C, the cooling output capacity can be enhanced by 2%. Increasing the cooling load production rate can improve the overall CPCC performance. Fig. 12 also shows that the cooling output capacity can be improved by raising the refrigerant flow rate entering the refrigeration cycle. At a constant cooling temperature, with a growth in the flow rate of the incoming increasing from 2.5 to 3 kg/s, the cooling output capacity grows by almost 21.6%.

**Fig. 13:**
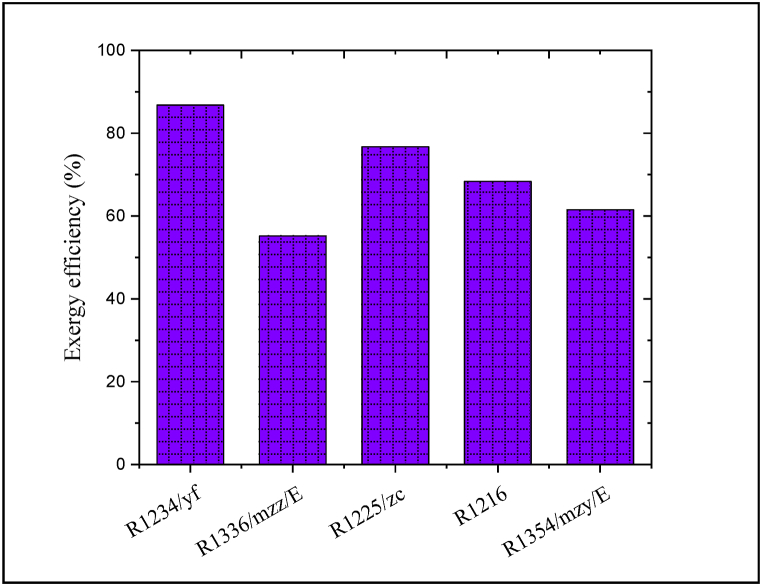
Exergy efficiency of the CPCC under the five considered refrigerants.

As discussed, among the five evaluated refrigerants, R1234/yf refrigerant could provide the best energetic behavior. The CPCC exergy efficiency was obtained under the five considered refrigerants and the results are depicted in Fig. 13. Because the proposed CPCC under R1234/yf refrigerant could produce the highest energy rate, therefore, the exergy efficiency under this refrigerant exhibits the highest value among other refrigerants. As expected, R1336/mzz/E and R1234/yf refrigerants result in the lowest and highest exergy efficiency of the proposed CPCC, respectively, such that, the calculated exergy efficiency for the proposed CPCC under R1234/yf refrigerant can be ∼31.6% higher compared to that R1336/mzz/E refrigerant.

### 4.1 Optimization outcomes

The maximum of the exergy efficiency and the minimum of the total cost under a two-objective optimization (based on genetic algorithm) are determined. The optimal outcomes are obtained under the optimal values of independent input variables. Based on this, the obtained outcomes from the two-objective optimization algorithm are presented in Table 8. As can be seen, the CPCC exergy efficiency is increased by 2.1% and at the same time the total cost is diminished by about 4.5%. To achieve such optimal findings, the refrigerant flow rate, cold temperature, and turbine inlet pressure should be increased by 8.8%, 33.72%, and 30%, respectively; while the turbine outlet temperature should be reduced by 7.6 ^°^C.

**Table 8:** Obtained outcomes from the two-objective optimization algorithm.

Parameters	Non-optimization	Optimization
Turbine outlet temperature (^°^C)	100.0	92.4
Refrigerant flow rate (kg/s)	2.5	2.72
Turbine inlet pressure (bar)	16.0	21.4
Cold temperature (^°^C)	8.0	10.4
Exergy efficiency (%)	86.78	88.61
Total cost (M USD)	0.269	0.257

### 4.2 Results comparison

Although cogeneration power and cooling cycles have been observed in the publications in many ways, the CPCC has a relatively new arrangement of components and structure that has not been comprehensively discussed and investigated from different standpoints. In most studies reported in the literature, the environmental assessment of such cogeneration cycles is not presented. Compared to the compression-based refrigeration process, an ejection-based refrigeration process can reduce energy consumption and consequently the system cost.

Additionally, due to the multi-level recovery, the environmental impacts are significantly reduced. The electricity cost of the proposed CPCC can be notably reduced due to the self-sufficiency in meeting the electricity requirements of the electric equipment. Besides that, there is an acceptable improvement in the cooling capacity rate of the energy cycle due to the energy losses decrement during the refrigeration process, which reduces the operating cost. Table 9 describes the comparison of the results. According to this table and literature survey, it can be concluded that the CPCC can offer a competitive process from thermodynamic, cost and environmental standpoints with other similar processes.

**Table 9:** Results comparison.

System model	Main results	Ref.
Multi evaporator vapor compression refrigeration cycle& An ORC unit	Exergy efficiency = 31.1%, COP value = 0.425	[93]
Compressor-driven ejector refrigeration cycle& An ORC unit	Under R141b: Exergy efficiency = 46.9%, COP value = 3.26	[94]
Combined cooling, heating and power cycle based on an ejector heat pump& An ORC unit	Under R141b/R134a: Power efficiency = 4.2%, COP value = 1.12	[95]
Organic Rankine flash cycle& An ejector integrated with a geothermal power generation cycle	Under R601: Net power = 634.14 kW, Exergy efficiency = 24.86%, Levelized energy cost = 0.477 USD/kWh	[74]
An ejector & An ORC unit& An energy storage unit	Exergy efficiency = 26%, Sum product unit cost = 13.72 USD/GJ	[51]
Two-stage ejector-expansion Transcritical refrigeration process& ORC unit	Under: Ethane: Exergy efficiency = 28.77%, COP value = 2.045	[96]
CPCC under a geothermal source& ORC& An ejector	Exergy efficiency = 59.2%, COP = 0.1224, cooling output = 93.73 kW	[56]
Hybrid organic flash cycle& An ejector	Under R600a/R601a: Efficiency = 22%, Exergy efficiency = 36.5%	[89]
Compression-based refrigeration cycle& An ORC unit	Under: R141b–R1234ze(Z), Exergy efficiency = 33%, COP value = 4.593	[97]
Compression-based refrigeration cycle& An ORC unit	Exergy efficiency = 14.97%, COP value = 0.44	[98]
Combined ORC unit and ejector-based refrigeration cycle to transformer and space cooling	Thermal efficiency = 26.2%, COP = 0.33, cooling output = 271.1 kW	[55]
An ORC unit& An ejector-based refrigeration system	Exergy efficiency = 22.62–32.3%, Sum unit cost of product = 45.79–58.87 USD/MWh	[53]
Three-level waste heat recovery plant based on An ORC system and an ejection-based refrigeration process	Under R1234/yf: Exergy efficiency = 86.78%, COP = 0.361, cooling output = 36.72 kW, CO2 reduction rate = 11,640 tons, Total cost = 0.269 M USD	Current study

## 5 Conclusions

The multi-aspect assessment and optimization of a cogeneration electricity and cooling load cycle was developed. The considered cogeneration process was based on a three-level waste heat recovery process based on an ORC unit and an ejection-based refrigeration process. Thermodynamic performance, cost feasibility and environmental analysis of the proposed process were comprehensively evaluated and discussed. A two-objective optimization was applied to minimize the total cost and maximize the exergy efficiency. Moreover, the comprehensive CPCC behavior is compared with a reference system (a single-level recovery/ORC process and a compression-based refrigeration process). The considered system's behavior was also examined under various low-GWP and zero-ODP refrigerants. Furthermore, the developed environmental analysis was based on two indicators: life cycle-climate performance and total equivalent-warming impacts. The proposed CPCC can achieve an exergy efficiency of approximately 86.8%; therefore, the CPCC exergy efficiency is improved by about 10.3% than the reference structure. Further, under the optimum case, the CPCC exergy efficiency can be increased by 2.1% and at the same time the total cost was declined by about 4.5%. Other important achievements are summarized as follows:

• R1234/yf is considered as the most suitable refrigerant that can lead to optimal results for both systems.
• The annual overall cost estimate of CPCC is equal to 0.269 M USD, which is more than 96% related to the capital and about 4% related to the operating cost. Due to the removal of the compressor and the decrement in the energy utilization and thermal duties on the one hand and the improvement of the heat recovery rate and the self-sufficiency of the proposed CPCC in electricity supply on the other hand, the investment cost of the suggested system is 21.4% lower than that reference cycle.
• About 11,640 tons of carbon dioxide can be reduced by using the ejector in the refrigeration process. Therefore, the proposed CPCC can address better environmental performance than the reference structure due to the use of low electricity consumption-equipment.

The proposed CPCC can offer a competitive process from thermodynamic, cost and environmental standpoints with other similar processes. However, it is recommended that an experimental prototype be evaluated before large-scale implementation. Although R1234/yf exhibited excellent performance, due to its flammability, it may cause problems in some industrial processes. Accordingly, it is recommended that this limitation be discussed and evaluated. In addition, the proposed CPCC can be integrated with the waste heat of fuel cells, which can be an interesting idea for subsequent works.

## Data availability statement

Data will be made available on request.

## CRediT authorship contribution statement

**Hima Nikafshan Rad:** Conceptualization, Formal analysis, Software, Writing – original draft. **Amir Ghasemi:** Writing – review & editing, Formal analysis, Investigation, Validation. **Mohammad Marefati:** Writing – original draft, Project administration, Methodology.

## Declaration of competing interest

The authors declare that they have no known competing financial interests or personal relationships that could have appeared to influence the work reported in this paper.
